# Gut commensal *Kineothrix alysoides* mitigates liver dysfunction by restoring lipid metabolism and gut microbial balance

**DOI:** 10.1038/s41598-023-41160-y

**Published:** 2023-09-06

**Authors:** Kyoung Jin Choi, Mi Young Yoon, Ji-Eun Kim, Sang Sun Yoon

**Affiliations:** 1 Department of Microbiology and Immunology, Yonsei University College of Medicine, 50-1 Yonsei-Ro, Seodaemun-Gu, Seoul, 03722 South Korea; 2 Brain Korea 21 Project for Medical Sciences, Yonsei University College of Medicine, Seoul, South Korea; 3 Severance Biomedical Science Institute, Yonsei University College of Medicine, Seoul, South Korea; 4 Institute of Immunology and Immunological Diseases, Yonsei University College of Medicine, Seoul, South Korea; 5 BioMe Inc., Seoul, South Korea

**Keywords:** Applied microbiology, Microbial communities

## Abstract

Metabolic dysfunction-associated steatotic liver disease (MASLD), previously known as Non-Alcoholic Fatty Liver Disease, is a widespread liver condition characterized by excessive fat buildup in hepatocytes without significant alcohol consumption. Manipulation of the gut microbiome has been considered to prevent and improve the occurrence and progression of MASLD, particularly through the gut-liver axis. This study aimed to investigate the correlation between the gut microbiome and liver function and determine whether the gut microbiome can ameliorate MASLD. We comparatively analyzed the gut microbiome composition between mice fed normal chow and those fed a high-fat diet and observed that the abundance of *Kineothrix alysoides* decreased in the high-fat group. Further analysis showed that treatment with *K. alysoides* in the high-fat diet group led to decreased weight loss, and MASLD attenuation. Importantly, *K. alysoides* treatment attenuated MASLD in mice fed a high-fat, high-fructose diet (HFHF), which can cause advanced liver damage. Furthermore, administration of *K. alysoides* altered the gut microbial composition in the HFHF diet group and improved MASLD. Overall, these findings demonstrate the potential of *K. alysoides* in restoring gut health and facilitating lipid metabolism to prevent and treat MASLD.

## Introduction

Metabolic dysfunction-associated steatotic liver disease (MASLD), previously known as Non-Alcoholic Fatty Liver Disease (NAFLD), is a widespread liver condition characterized by excessive fat buildup in hepatocytes without significant alcohol consumption. The renaming to MASLD reflects its diverse metabolic components and associated health risks beyond the liver. Its prevalence has escalated worldwide, coinciding with the rise in obesity, sedentary lifestyles, and metabolic disturbances, posing significant challenges to public health systems. Approximately 25% of the global population is affected, with higher rates in Western countries^1,2^. Alarmingly, MASLD is now increasingly diagnosed in children and adolescents, emphasizing the need for early prevention and intervention strategies^3^.

Understanding the complex mechanisms underlying MASLD is essential for identifying potential therapeutic targets. The multifactorial nature of MASLD necessitates a comprehensive approach to address its diverse etiological factors. Therapeutic interventions can target various aspects, such as hepatic lipid metabolism, inflammation, oxidative stress, gut microbiome, and insulin resistance^4,5^. Research into these areas aims to develop effective treatment options for managing MASLD and its associated complications.

The gut-liver axis is a bidirectional communication pathway between the gut and the liver, playing a crucial role in various aspects of health and disease. This axis involves a complex interplay between the gut microbiome, gut epithelium, immune system, and the liver, with the potential to influence liver function, metabolism, and overall health^6,7^. The gut microbiome plays a pivotal role in the breakdown and fermentation of dietary nutrients, producing metabolites that can have beneficial effects on the host. One of the key roles of the gut-liver axis is to maintain intestinal barrier integrity. A healthy gut microbiome helps to support the gut barrier, preventing the translocation of harmful substances and bacteria from the gut into the bloodstream. When the gut barrier is compromised, as seen in conditions like dysbiosis (an imbalance of gut microbial communities), harmful bacteria and their products can enter the bloodstream and reach the liver through the portal vein^8^.

In the liver, the presence of these harmful substances triggers an immune response and inflammation. This inflammatory response can contribute to the development of liver diseases, including hepatic steatosis (fatty liver), non-alcoholic steatohepatitis (NASH), and eventually lead to Metabolic dysfunction-associated steatotic liver disease (MASLD). Moreover, the gut-liver axis can also affect systemic metabolism, insulin sensitivity, and lipid metabolism, further influencing the development and progression of liver diseases^6,7,9^.

In the context of MASLD, there is growing interest in understanding how specific gut microbes impact liver health. By modulating the gut microbiome, researchers aim to discover potential therapeutic approaches to improve liver function and reduce the impact of liver diseases^10–12^. Interventions that target the gut-liver axis and the gut microbiome offer promising opportunities for the development of personalized and targeted therapies for managing MASLD.

In particular, differences in intestinal microbial communities between healthy and obese individuals have demonstrated the involvement of intestinal microorganisms in fat metabolism. Microbial community analyses in high-fat (HF)-fed mice have revealed that the ratio of *Firmicutes*/*Bacteroidetes* is elevated in obese mice^9,13–16^; similar results have been obtained in humans. Several studies have demonstrated that *Bacteroides* spp. are closely associated with MASLD. The most common commensal intestinal bacterial species include *Bacteroides xylanisolvens*, *Bacteroides uniformis*, *Bacteroides thetaiotaomicron*, and *Bacteroides vulgatus*. The presence of *B*. *xylanisolvens* in the intestinal tract of MASLD-attenuated rats was positively correlated with reduced MASLD progression^16^. *B. uniformis* has been shown to reduce cholesterol and triglyceride levels in obese mice^17^ and alleviate hepatic steatosis. *B. thetaiotaomicron* can reduce body weight and lipogenesis-related gene expression^18^. *B. vulgatus* can attenuate atherosclerosis by adjusting intestinal lipopolysaccharide (LPS) levels^19^.

Therefore, in this study, we aimed to identify less well-known bacterial stains that may have potential therapeutic effects on MASLD. By uncovering the link between specific gut microbes and their influence on liver function, we aim to contribute to the development of novel and targeted therapies for managing MASLD.

## Results

### High-fat intake reduces the abundance of *Kineothrix* and *Turicibacter*

**Figure 1:**
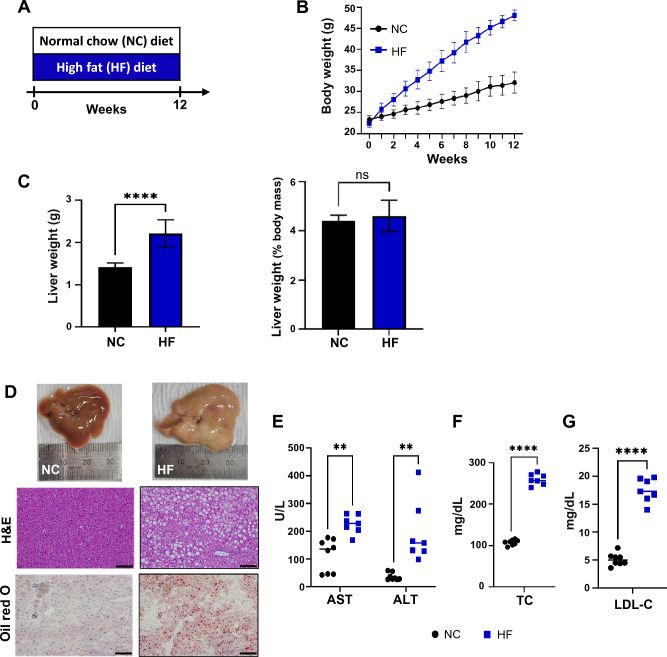
Effects of high-fat diet on liver function. (**A**) Mice were fed normal chow (NC, n = 8 mice) and a high-fat diet (HF, n = 7 mice) for 12 weeks. (**B**) Body weight was measured throughout the 12-week period. (**C**) Liver weight and liver-to-body mass ratio were measured after 12 weeks. (**D**) Histological analysis of liver tissues stained with hematoxylin and eosin (H&E) and with Oil Red O (scale bars, 100 μm). (**E**–**G**) Biochemical parameters, including aspartate transaminase (AST), alanine transaminase (ALT), serum total cholesterol (TC), and serum low-density lipoprotein cholesterol (LDL-C) were measured. Data are presented as means ± SD. Statistical analysis was performed using a one-way analysis of variance. *p < 0.05; **p < 0.01; ***p < 0.001; ****p < 0.0001.

To investigate the effect of a high-fat (HF) diet on the gut microbiome and liver, we compared the gut microbial composition before and after feeding the HF diet. We fed male C57BL/6 specific pathogen-free (SPF) mice (8-week-old) an HF diet for 12 weeks (Fig. 1A). During this 12-week period, both body weight significantly increased in mice fed the HF diet compared to those fed normal chow (NC) (Fig. 1B,C). The primary histological features of MASLD include hepatocellular ballooning, fat accumulation, and steatosis^20,21^. To assess the effect of HF diet on hepatocytes, we conducted histological analyses using hematoxylin and eosin (H&E) and Oil Red O staining. The HF mice showed hepatocyte ballooning and fat deposition (Fig. 1D). Moreover, aspartate transaminase (AST) and alanine transaminase (ALT), considered indicators of liver damage, increased significantly by 2.0-fold and 5.3-fold, respectively, in HF mice (Fig. 1E). Additionally, serum total cholesterol (TC) increased 2.3-fold (Fig. 1F), and low-density lipoprotein (LDL) cholesterol levels were significantly higher (3.4-fold) in HF mice than in NC mice (Fig. 1G). Based on the histological features and their indicators, we confirmed that feeding an HF diet for 12 weeks could induce MASLD in mice.

**Figure 2:**
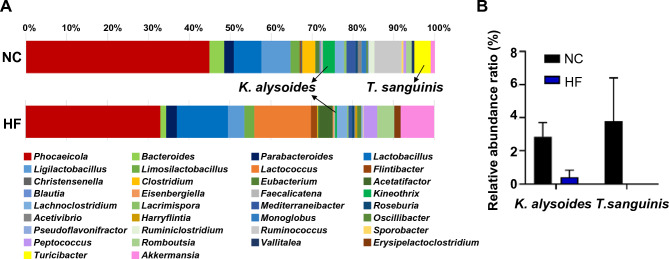
A high-fat diet alters the gut microbiome composition. Microbiome populations were analyzed at the genus and species levels among different groups. Fecal samples were collected at the end of the high-fat diet, and DNA was extracted for 16S rRNA amplicon sequencing. (**A**) The microbiome populations at the genus level are represented by bars. Green and yellow represent *Kineothrix* and *Turicibacter*. (**B**) Species levels of *Kineothrix* and *Turicibacter*. Black represents the normal chow-fed group (NC) and blue represents the high-fat-fed group (HF). Data are presented as mean ± SD.

Next, we compared the gut microbiome composition of NC and HF mice to identify the gut microbes that could affect MASLD. At the phylum level, similar to other studies^9,13–16^, the ratio of *Firmicutes* to *Bacteroidetes* was 1.6-fold higher in the HF mice than in the NC mice. The relative abundance of gut bacteria in each group is shown in Fig. 2A. At the genus level, mice fed an HF diet had an increased relative abundance of *Lactobacillus* (6.55% changed to 12.05%), *Lactococcus* (0–13.28%), and *Akkermansia* (0.96–7.97%) and a decreased relative abundance of *Phocaeicola* (43.29%–31.76%), *Bacteroides* (3.45%–1.38%), *Ligilactobacillus* (6.84%–3.93%), and *Ruminococcus* (6.33%–0.10%) compared to those in the NC group. Among bacteria that exhibited changes in their composition rate in the gut, we selected *K. alysoides* and *T. sanguinis* whose rates were lowered from 2.86% to 0.42% and from 3.78% to 0%, respectively, in the HF group compared to the NC group (Fig. 2B); notably, few studies have reported the roles of these bacteria^16,22^.

### Effects of *K. alysoides* on high-fat diet-induced mice

A recent study reported that *K. alysoides* increased 56.6-fold in the gut of MASLD-alleviated mice compared with that in vehicle-treated mice^16^. In an atherosclerosis model using a HF diet, berberine, a naturally occurring alkaloid, showed an anti-atherosclerotic effect that altered the composition of gut microbes, wherein *Turicibacter* was enriched^22^. To assess the effectiveness of *K. alysoides* (Ka) and *T. sanguinis* (Ts) in mitigating MASLD symptoms, we administered Ka or Ts to mice via oral gavage after they were fed either an NC or HF diet for 8 weeks (Fig. S1A). Contrary to our expectations, mice fed Ka along with the HF diet experienced some relief in weight gain, whereas mice fed the Ts and HF diets gained weight (Fig. S1B). Furthermore, although the HF diet induced a substantial increase in body weight, liver damage indicators (AST and ALT) and LDL levels, except for TC, were not significantly aggravated, regardless of the presence of bacteria or sterile 1 × phosphate-buffered saline (PBS) (Fig. S1D,E). These results indicate that the HF diet-induced MASLD model is not suitable for the assessment of the potential of Ka to ameliorate MASLD symptoms. Consequently, we decided to further investigate the role of Ka in MASLD using a different well-established model that showed clear pathological features.

### *K. alysoides* reduces lipid accumulation in the liver

**Figure 3:**
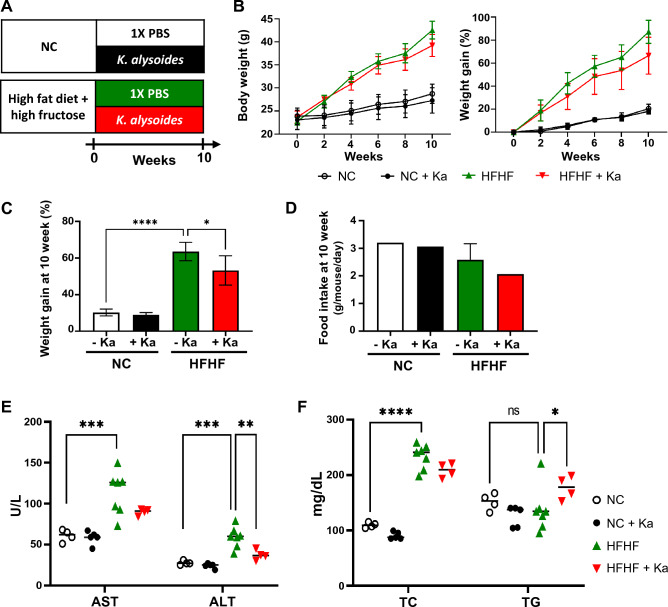
Effects of *K. alysoides* on body weight gain in mice fed high-fat, high-fructose (HFHF) diet. (**A**) Normal chow (NC)-fed mice and high-fat, high-fructose (HFHF)-fed mice were treated daily with 1X phosphate buffered saline (PBS) or *K. alysoides* (Ka) by oral gavage for 10 weeks. (**B**) Body weight and weight gain percentage were measured once a week for 10 weeks. The rate of weight gain (**C**) and food intake (**D**) at 10 weeks were indicated, separately. (**E**,**F**) Biochemical parameters, including aspartate transaminase (AST), alanine transaminase (ALT), serum total cholesterol (TC), and triglyceride (TG) were measured. Data are presented as means ± SD. Statistical analysis was performed using a one-way analysis of variance. *p < 0.05; **p < 0.01; ***p < 0.001; ****p < 0.0001.

Dietary fructose administered with fat accelerates liver damage and can induce severe metabolic disease^23,24^. Studies have also shown that mice fed a high-fructose diet exhibit significant MASLD symptoms^25,26^. Thus, to obtain a clear evaluation of the impact of Ka on liver health, we utilized a MASLD mouse model induced by a high-fat and high-fructose (HFHF) diet. We administered Ka to mice via oral gavage, with either HFHF or NC diet, for 10 weeks and measured body weight and food intake once a week (Fig. 3A). The body weight of the mice fed the NC diet and Ka did not change significantly compared to that of the mice fed the NC diet without Ka. However, administering Ka to mice while on the HFHF diet led to a 11.04% reduction (*p-value* = 0.013) in weight gain compared to that of the HFHF diet without Ka (Fig. 3B,C). This effect was likely caused by the reduced food intake in the Ka-treated HFHF diet group (Fig. 3D). Next, we investigated the serological indicators (Fig. 3E,F) and found that administering Ka with HFHF diets decreased the serum AST and ALT levels by 15.97% (*p* = 0.166) and 31.19% (*p* = 0.004), respectively, compared with those in the HFHF diet without Ka treatment. Contrary to our expectations, there was no change in the TC levels, and the total glycerol (TG) levels in the serum were higher in the Ka-treated HFHF diet group than in the HFHF diet group without Ka (Fig. 3F).

**Figure 4:**
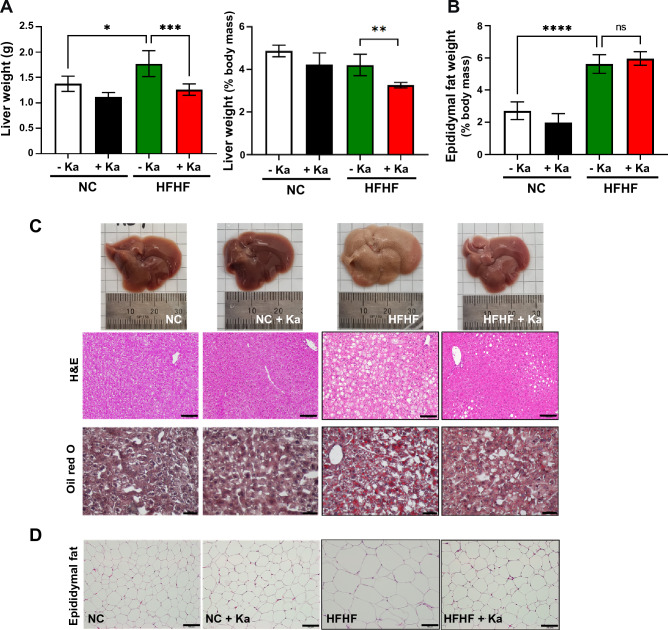
Effects of *K. alysoides* on lipid accumulation in the liver of mice fed a high-fat, high-fructose (HFHF) diet. (**A**,**B**) Liver weight, liver-to-body mass ratio, and epididymal fat-to-body mass ratio were measured at the end of the experiment. (**C**) Representative images of liver tissues stained with hematoxylin and eosin (H&E) (scale bars, 100 μm) and Oil Red O (scale bars, 50 μm) are shown. (**D**) Histological analysis of epididymal fat stained with H&E (scale bars, 100 μm) is also shown.

Notably, the liver weight did not show any significant difference among the NC-fed groups, whereas in the Ka-treated group fed the HFHF diet, the liver weight decreased significantly by 27.78% compared to that in the HFHF group without Ka (Fig. 4A). Importantly, this decreased effect was evident even after adjusting for body weight. However, there was no significant difference in epididymal fat mass between the NC and HFHF groups with respect to Ka administration (Fig. 4B). The livers of mice fed the HFHF diet displayed a paler, enlarged appearance, and numerous lipid droplets, indicating fat accumulation inside the hepatic cells, compared to that in the NC group (Fig. 4C). Interestingly, the administration of Ka along with an HFHF diet reversed the liver phenotype to that observed in the NC group. H&E and Oil Red O staining showed a reduction in hepatocyte ballooning and fat deposition in the livers of mice fed a HFHF diet and treated with Ka (Fig. 4C). Moreover, lipid droplet size in the epididymal fat of HFHF-fed mice with Ka also decreased (Fig. 4D). These findings demonstrated that *K. alysoides* has the potential to limit fat accumulation caused by the HFHF diet in the liver.

### *K. alysoides* treatment impacts the gut microbiome

**Figure 5:**
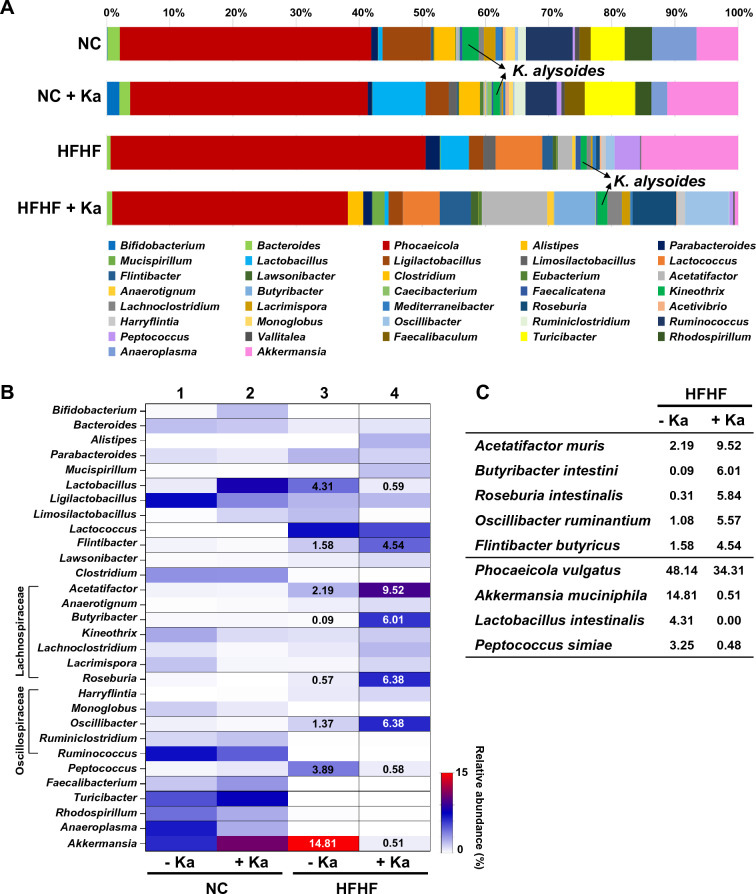
Comparison of gut microbiome composition between the *Kineothrix alysoides* (Ka) treated- and non-treated groups. (**A**) Microbial compositions at the genus level in mice fed with the normal chow (NC) or high-fat, high-fructose (HFHF) diet with or without *K. alysoides* (Ka) are shown in bar plots. Green color represents *Kineothrix*. (**B**) The major microbes with a relative abundance of at least more than 1% in all groups are shown. A heatmap generated using GraphPad Prism displays the relative abundance of the relevant genus. The degree of red or white color indicates high or low abundance in each group. Several values of relative abundances are shown within the heatmap. Columns 1 and 2 represent the NC group, while columns 3 and 4 represent the HFHF group. (**C**) The relative abundance values of representative species that were significantly altered in mice fed the HFHF diet and administered with Ka are shown. Data are presented as the mean. NC, normal chow diet; NC + Ka, normal chow diet with Ka; HFHF, high-fat and high-fructose diet; HFHF + Ka, high-fat and high-fructose diet with Ka.

Dysbiosis is a notable environmental risk factor that contributes considerably to MASLD progression^7,27,28^. Modifying the gut microbiome with probiotics has the potential to alleviate gut microbiome-associated diseases^16,29,30^. To investigate the association between *K. alysoides* treatment and the gut microbiome, stool samples were collected and summed from each of the groups. The 16S rRNA amplicon sequencing results (Fig. 5A) revealed that mice fed the HFHF diet had a higher relative abundance of *Phocaeicola, Akkermansia*, *Lactococcus*, *Lactobacillus*, and *Peptococcus* and a lower abundance of *Ligilactobacillus*, *Ruminococcus*, *Anaeroplasma*, *Turicibacter*, *Rhodospirillum*, *Clostridium*, and *Kineothrix* compared to those in the NC group.

To briefly present the dominant gut microbiome of all groups, we summarized the relative abundance of dominant bacteria, excluding *Phocaeicola* (Fig. 5B). Interestingly, HFHF-fed mice showed alterations in some genera after *K. alysoides* treatment. The relative abundances of *Acetatifactor*, *Roseburia*, *Oscillibacter*, *Butyribacter*, and *Flintibacter were* increased in mice fed the HFHF diet and treated with Ka compared to those in the group without Ka. Especially at the species level, the abundance of minor microbes, including *Acetatifactor muris*, *Butyribacter intestini*, *Roseburia intestinalis*, *Oscillibacter ruminantium*, and *Flintibacter butyricus*, increased to 9.52%, 6.01%, 5.84%, 5.57%, and 4.54%, respectively, in the mice fed with HFHF and Ka (Fig. 5C). Among the bacteria showing increased abundance, *Roseburia,* reportedly attenuates alcoholic fatty liver disease in a mice model^30^. The Ka treatment group in the HFHF diet showed a decreased relative abundance of *Phocaeicola vulgatus*, *Akkermansia muciniphila*, *Lactobacillus intestinalis*, and *Peptococcus simiae* compared with that in the HFHF group without Ka (Fig. 5C). These results suggest that *K. alysoides* treatment increases its levels as well as the abundance of other microorganisms involved in liver disease amelioration.

### *K. alysoides* regulates lipid metabolism and inflammation

A previous study has revealed that *K. alysoides* is one of the most abundant bacteria in the human gut microbiome and can produce butyrate, which has potential therapeutic effects^31,32^. To verify the role of *K. alysoides* in butyrate production, we analyzed the levels of short-chain fatty acids (SCFAs) in the guts of all groups (Fig. S2). Of note, the levels of acetic acid, butyric acid, and propionic acid were reduced in the HFHF-fed group compared to the control group fed the NC diet (Fig. S2A–C). Contrary to our expectations, Ka treatment did not significantly affect SCFA concentrations in mice fed the HFHF diet, although the production of butyric acid and propionic acid was increased by Ka treatment during NC feeding (Fig. S2B,C).

**Figure 6:**
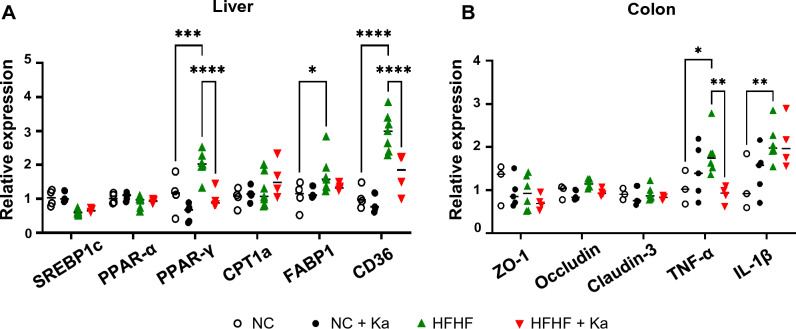
Effects of *Kineothrix alysoides* on the gene expression levels in the liver and colon in high-fat, high-fructose (HFHF)-induced models. (**A**) Relative mRNA expression levels in the liver of mice treated with *K. alysoides* (Ka) or PBS were determined using qRT-PCR for SREBP1c, PPAR-α, PPAR-γ, CPT1a, FABP1, and CD36. Expression levels were normalized to GAPDH and expressed as fold changes compared with those in the normal chow-fed mice. (**B**) Relative mRNA expression levels in the colon of mice treated with Ka or PBS were determined using qRT-PCR for tight junction and inflammatory markers, including ZO-1, Occludin, Claudin-3, TNF-α, and IL-1β. Expression levels were normalized to those of GAPDH and expressed as fold changes compared with those in normal chow-fed mice. Data are presented as means ± SD. Statistical analysis was performed using one-way analysis of variance. *p < 0.05; **p < 0.01; ***p < 0.001; ****p < 0.0001. SREBP1c, sterol regulatory element-binding protein 1c; PPAR, peroxisome proliferator activated receptor; CPT1a, carnitine palmitoyltransferase 1; FABP1, fatty acid binding protein; ZO-1, Zonula occludens-1; TNF-α, tumor necrosis factor-alpha; GAPDH, glyceraldehyde 3-phosphate dehydrogenase.

To explore other factors influenced by Ka treatment, we examined the gene expression levels of pathways related to MASLD in the liver and colon. As shown in Fig. 6A, the HFHF diet induced the expression of genes related to lipogenesis and fatty acid uptake pathways, including PPAR-γ (1.78-fold), FABP1 (1.58-fold), and CD36 (2.9-fold). Although genes related to de novo fatty acid synthesis and lipid oxidation, such as SREBP1c and PPAR-α, were not affected, the administration of Ka to HFHF-fed mice significantly suppressed the expression levels of PPAR-γ (by 1.58-fold) and CD36 (by 1.86-fold) compared to those in HFHF mice.

Furthermore, we investigated the beneficial effects of Ka treatment in the colon by comparing the expression levels of genes encoding tight junction proteins (ZO-1, Occludin, and Claudin-3) and cytokines (TNF-α and IL-1β) (Fig. 6B). No significant differences were observed in the expression levels of genes related to tight junction proteins. However, the gene expression level of TNF-α was significantly decreased by 1.94-fold in HFHF-fed mice treated with Ka compared with that in HFHF-fed mice without Ka (Fig. 6B). Our results indicate that Ka regulates genes involved in fat deposition in HFHF-fed mice by suppressing genes related to lipogenesis and fatty acid uptake in the liver. Furthermore, we inferred it regulates colonic inflammation by reducing the expression of proinflammatory cytokine genes.

## Discussion

MASLD (Metabolic dysfunction-associated steatotic liver disease), known as non-alcoholic fatty liver disease (NAFLD), has emerged as a significant global health concern, closely linked to the rise in obesity and metabolic disorders^33,34^. Excessive dietary fat and simple carbohydrates can enter the liver through the uptake of fatty acids and de novo lipogenic pathways^35,36^. They are converted to triglycerides, which are stored as lipid droplets in the liver^35^. When the body requires energy, stored triglycerides are broken down into fatty acids, which are then exported from the liver^37^. An imbalance in lipid storage and metabolism can lead to MASLD^37,38^. Recently, liver health and the gut microbiome have been discovered to be connected through the “gut-liver axis”^39,40^. This axis plays a crucial role in various aspects of health and disease, including metabolic disorders like MASLD. Dysbiosis, caused by a diet high in simple carbohydrates, can disrupt the gut barrier, leading to inflammation and an increased risk of liver disease^41^. Understanding the interactions between the gut microbiome, liver function, and lipid metabolism is vital for identifying potential therapeutic targets for MASLD. Our findings indicate that *K. alysoides* can mitigate lipid accumulation in the liver by inhibiting fatty acid absorption and restoring balance in the gut microbiome.

The gut microbiome is closely related to lipid metabolism and contributes to the expression of regulatory factors in the host, necessitating the understanding of its role in various metabolic diseases^42,43^. Mounting evidence has demonstrated that gut microbiome alterations influence the gut-liver axis and are related to the progression of liver diseases such as cirrhosis and MASLD^29,44–46^. The patients with MASLD exhibit specific changes in gut microbiome composition, characterized by an increased abundance of certain *Firmicutes* species, such as Clostridium and Streptococcus, and a decrease in some *Bacteroidetes* species, including *Bacteroides thetaiotamicron* and *Bacteroides vulgatus*^9,14–16^. Consequently, several strategies have been tested to improve gut microbiome composition and alleviate MASLD, including the use of probiotics and prebiotics to promote beneficial microorganisms and reduce harmful ones^16,17,29,45,47–49^.

In a relevant study, Qiao et al. found that an HF diet decreased *Bacteroides*, which produces folate; however, treatment with *B. xylanisolvens* and Ganoderma meroterpene derivative (GMD) improved liver function^16^. In particular, GMD altered *K. alysoides* abundance by up to 57.5-fold. Qiao et al. suggested that the increased presence of *K. alysoides* might enhance gut barrier function, leading to a reduction in endotoxemia, as evidenced by decreased levels of plasma lipopolysaccharide and TNF- α^16^. These findings indicated a potential role for *K. alysoides* in mitigating lipid accumulation in the liver by promoting gut barrier integrity and reducing the systemic inflammation triggered by endotoxemia.

In our study, we observed the decreased gene expression levels of TNF-α, in the colon and PPAR-γ, CD36 in the liver of mice fed the HFHF diet after administration of *K. alysoides*. Additionally, the levels of ALT, a marker of liver injury^50^, were significantly decreased following Ka administration. These results further suggest a potential relationship between *K. alysoides* and inflammation, offering valuable insights into its therapeutic potential for mitigating liver-related pathologies.

Furthermore, a previously published study demonstrated the alleviating effects of *R. intestinalis* on alcoholic fatty liver and suggested that these effects were associated with a significant decrease in PPAR-γ and CD36 levels^30^. Similarly, in our study, we observed a significant decrease in PPAR-γ and CD36 levels in the group treated with *K. alysoides*, which exhibited alleviation of fatty liver. Based on these findings, we hypothesize that changes in gene expression related to triglyceride synthesis and fatty acid uptake may directly influence liver damage.

*K. alysoides* has been identified as a butyrate-producing microbe, which is important for maintaining gut health because of its anti-inflammatory and immune-regulating properties, and for maintaining the gut barrier^31^. In this study, *K. alysoides* treatment with an HFHF diet increased the abundance of *Lachnospiraceae* and *Oscillospiraceae* (Fig. 5B), which are known for their ability to produce SCFAs, especially butyric acid^51^. However, Ka administration did not increase butyric acid levels (Fig. S2B). Among the microbes whose abundance changed upon Ka treatment in the HFHF diet (Fig. 5C), *R. intestinalis* was found to ameliorate liver inflammation and fibrosis, thus playing a crucial role in maintaining the gut barrier^30^. Conversely, low *R. intestinalis* counts are associated with various health conditions^52^. *B. intestini* and *A. muris* (Fig. 5C) can also produce butyrate and have been linked to the prevention of metabolic disorders^53,54^. *O. ruminantium* is involved in SCFA production and has anti-inflammatory effects^55–57^, but its role in the gut remains unclear. Although the abundance of *P. vulgatus* decreased with Ka treatment, it remained the most abundant microbe in the gut of mice treated with Ka and HFHF diets. Studies have reported that *P. vulgatus* has lipid-lowering effects that protect against alcohol-induced or metabolic liver disease^19,58^. *A. muciniphila* has both positive and negative roles in the gut, producing SCFAs with anti-inflammatory properties but also potentially increasing gut permeability^59^. Collectively, the effect of Ka treatment on liver health is likely attributable to changes in the gut microbiome composition and their complicated relationships with each other, rather than simply the production of SCFAs.

We evaluated the efficacy of *K. alysoides* in reducing liver damage caused by HFHF diet-induced MASLD^60–65^. Although the degree of liver damage is less severe, an HFHF diet is commonly used to model human MASLD^21,66^. Fructose is metabolized in the liver to fructose-1-phosphate by fructokinase C, leading to intracellular ATP depletion and uric acid production, which have harmful effects on the liver, such as increased triglyceride accumulation and inflammasome activity^61^. Excessive fructose intake can also activate hepatic de novo lipogenesis and impair intestinal barrier function, thereby increasing bacterial metabolite translation^67,68^. Studies have shown that increased fructose intake is a critical risk factor for MASLD progression, which affects liver and gut metabolism^23^. Therefore, the HFHF model allowed us to confirm the effectiveness of Ka more specifically in MASLD than in the HF diet (Figs. S1 and 3). Administering Ka with HFHF decreased body weight gain and the primary histological features of MASLD (Fig. 4C), but did not significantly affect the levels of serological indicators such as AST and TC (Fig. 3E,F). Interestingly, the serum level of TG in mice on the HFHF diet and administered Ka was significantly increased compared to that in mice fed HFHF without Ka (Fig. 3F). We speculate that this increase may be attributed to the lipid being released from the liver of HFHF- and Ka-fed mice, translocating into the circulation. However, this warrants verification, such as investigation of the lipid metabolism pathway. Furthermore, to further confirm the relationship between Ka and other gut microbes whose abundances were changed, as well as the mechanism underlying the ameliorative effect of Ka in MASLD, it is necessary to verify these findings over a longer duration of the HFHF diet (> 12 weeks).

In conclusion, we investigated the potential of *K. alysoides* in the treatment of MASLD. By targeting the decreased abundance of microbial strains following HF feeding, we found that *K. alysoides* could alleviate liver damage to be able to reduce intestinal inflammation. The reduced fat accumulation in the liver and lower intestinal inflammation may be linked to changes in the gut microbiome. Overall, our findings suggest that *K. alysoides*, a commensal microbe, can prevent the progression of fatty liver disease and has the potential to be developed as a Live Biotherapeutic Product (LBP) for the treatment of MASLD.

## Materials and methods

### Animal experiments

We used 7-week-old male C57BL/6 mice were purchased from Orient Bio (Sung-nam, Korea). The mice were provided with sterile water and food. All experiments involving mice were conducted in accordance with the guidelines of the Department of Animal Resources of the Yonsei Biomedical Research Institute and regulations within ARRIVE (Animal Research: Reporting of In Vivo Experiment) guidelines. This study was approved by the Committee on the Ethics of Animal Experiments at Yonsei University College of Medicine (permit numbers, 2021-0139).

After an adaptation period of one week, the mice were fed either an NC (13.12% of energy from fat, PicoLab Rodent Diet 20, LabDiet 5053, Texas) or a high-fat diet (HF, 60% of energy from fat, TD.06414, ENVIGO, USA) for 12 weeks. Mice were randomly divided into two groups: NC- and HF-fed (n = 7 and n = 8, respectively).

A schematic of the animal experiments using a high-fat diet with 30% fructose-supplemented drinking water (HFHF) is shown in Fig. 3A. Mice were fed either NC or HFHF for 10 weeks. The mice were then randomly divided into four groups: NC control mice (n = 4); NC + Ka, mice fed NC and *K. alysoides* (n = 5); HFHF mice (n = 7); and HFHF + Ka, mice fed an HFHF diet and *K. alysoides* (n = 4).

Before the sacrifice in the end of the experiment, mice from each group were placed in empty cages. After 20 min, fecal samples remaining in the cages were collected and pooled into individual tubes for each group. For HF model, we performed fecal pooling of two samples from two cages within each group. For the HFHF model, we summed the fecal samples of each group. The obtained fecal samples were stored in a deep freezer until microbial composition analysis was performed. The mice from which fecal samples were collected were euthanized in a CO_2_ chamber, and blood and tissues were obtained. Blood was collected first, followed by the preparation of liver, cecum, and colon tissues in that order. Each tissue was placed in a cryotube to prevent RNA degradation and immediately immersed in liquid nitrogen.

### Bacteria cultivation, preparation, and treatment

*K. alysoides* DSM 100556 was purchased from the Leibniz Institute, DSMZ-German Collection of Microorganisms and Cell Cultures (Braunschweig, Germany). All anaerobic culture media were deoxygenated for 48 h before use, and bacteria were cultured at 37 °C in an anaerobic chamber with mixed anaerobic gas (5% carbon dioxide, 5% hydrogen, and 90% nitrogen). Tryptic soy broth (TSB; cat. No. 211825, BD Difco Tryptic Soy Broth, BD, USA) supplemented with 0.5% cellobiose (cat. C0056; TCI, D-( +)-cellobiose, Japan) were used to cultivate *K. alysoides*. All *K. alysoides* cultures were conducted in an anaerobic chamber. During the growth phase, we assessed the colony-forming units (CFU) of Ka cells and plotted the CFU values against the optical density (OD). The harvested cells at each growth phase were measured for OD at 600 nm. Subsequently, the cells were washed with sterile 1 × phosphate-buffered saline (PBS) and serially diluted by tenfold. The diluted cells were spread on TSB with 0.5% cellobiose plates and incubated within the anaerobic chamber. To prepare the strains for oral gavage, we calculated the cells to be 10^10^ CFU/200 μL based on OD values obtained from the CFU-OD plot. The Ka cells were aliquoted within the chamber before the treatment to mice. Additionally, we re-confirmed the CFU of the cells using the same method as described above.

### Serum biochemical analysis

Blood was extracted from the posterior vena cava of each mouse using a sterile 1 mL syringe. Serum was separated from the blood by centrifuging the sample at 3,000 rpm at 4 °C for 15 min. AST, ALT, TC, TG, and LDL-C levels were quantified by DooYeol Biotech (Seocho-gu, Korea) and Department of Laboratory Animal Resources (Yonsei Biomedical Research Institute, Yonsei University College of Medicine).

### Histological analysis

Liver tissues and epididymal fat were fixed in 10% formalin solution. Frozen liver tissues were sliced into 4 μm-thick sections and stained with Oil Red O. The paraffin blocks of the liver tissue and epididymal fat were sliced into 4 μm-thick sections and stained with hematoxylin and eosin (H&E). Stained sections were visualized under an optical microscope (BX53M; Olympus). Histological analyses were performed by Tego Science (Gangseo-gu, Korea) and Department of Laboratory Animal Resources (Yonsei Biomedical Research Institute, Yonsei University College of Medicine).

### RNA extraction and quantitative real-time PCR assay

Liver and colon tissues were stored at −80 °C before RNA extraction. RNA was extracted from frozen tissue samples using TRIzol reagent (Invitrogen, USA). Complementary DNA was synthesized from 2 μg of RNA using PrimeScript Reverse Transcriptase (Takara, Japan). The qRT-PCR primer sequences are listed in Table S1. Real-time PCR was performed using the PowerUP SYBR Green Master Mix (Thermo Fisher Scientific). The measured mRNA levels were normalized to those of glyceraldehyde 3-phosphate dehydrogenase and expressed as fold changes relative to those of the control group.

### Microbiome composition analysis

Fecal sample DNA was extracted using a DNeasy PowerSoil Kit (Qiagen, Hilden, Germany) according to the manufacturer's instructions. The extracted DNA was quantified using a Quant-IT PicoGreen (Invitrogen). Sequencing libraries were prepared according to Illumina 16S Metagenomic Sequencing Library protocols to amplify the V3 and V4 regions. The input of 2 ng of gDNA was PCR-amplified with 5X reaction buffer, 1 mM dNTP mix, 500 nM of each of the universal F/R PCR primers, and Herculase II fusion DNA polymerase (Agilent Technologies, Santa Clara, CA). The cycle conditions for the 1st PCR were 3 min at 95 °C for heat activation, 25 cycles of 30 s at 95 °C, 30 s at 55 °C, and 30 s at 72 °C, and a 5 min final extension at 72 °C. The universal primer pair sequences with an Illumina adapter overhang used for the first amplification were as follows: V3-F:5′-TCG TCG GCA GCG TCA GAT GTG TAT AAG AGA CAG CCT ACG GGN GGC WGC AG -3′, V4-R:5′- GTC TCG TGG CTC GGA GAT GTG TAT AAG AGA CAG GAC TAC HVG GGT ATC TAA TCC -3′. The 1st PCR product was purified by using AMPure beads (Agencourt Biosciences). Following purification, 2 μL of the 1st PCR product was PCR-amplified for final library construction containing the index using the NexteraXT Indexed Primer. The cycle conditions for the 2nd PCR were the same as those for the 1st PCR, except that 10 cycles were used for the amplification. PCR products were purified using AMPure beads. The final purified product was quantified using qPCR according to the qPCR Quantification Protocol Guide (KAPA Library Quantification kits for Illumina Sequencing platforms) and qualified using TapeStation D1000 ScreenTape (Agilent Technologies, Waldbronn, Germany). Paired-end (2 × 300 bp) sequencing was performed by Macrogen on a MiSeq platform (Illumina, San Diego, CA, USA).

### Statistical analysis

Data are expressed as means ± standard deviation (S.D.). All data calculations and statistical analyses were performed using the GraphPad Prism ver. 9.4.1 (GraphPad Software Inc., La Jolla, CA, USA). Differences between groups were analyzed using one-way analysis of variance, and *p* values < 0.05 were considered statistically significant. *p < 0.05; **p < 0.01; ***p < 0.001; ****p < 0.0001.

### Supplementary Information

Supplementary Information 1.

Supplementary Information 2.

## Data Availability

All raw sequences have been archived in Zenodo. DOI: 10.5281/zenodo.8054022. (https://zenodo.org/record/8054022).
