# Supplementary material for: Gut commensal Kineothrix alysoides mitigates liver dysfunction by restoring lipid metabolism and gut microbial balance
Source: Sci Rep. 2023 Sep 6;13:14668. doi: 10.1038/s41598-023-41160-y (PMC10482948; doi:10.1038/s41598-023-41160-y)
Supplement: Supplementary file 1 — Supplementary Information 1. [file 41598_2023_41160_MOESM1_ESM.pptx]

## Slide 1
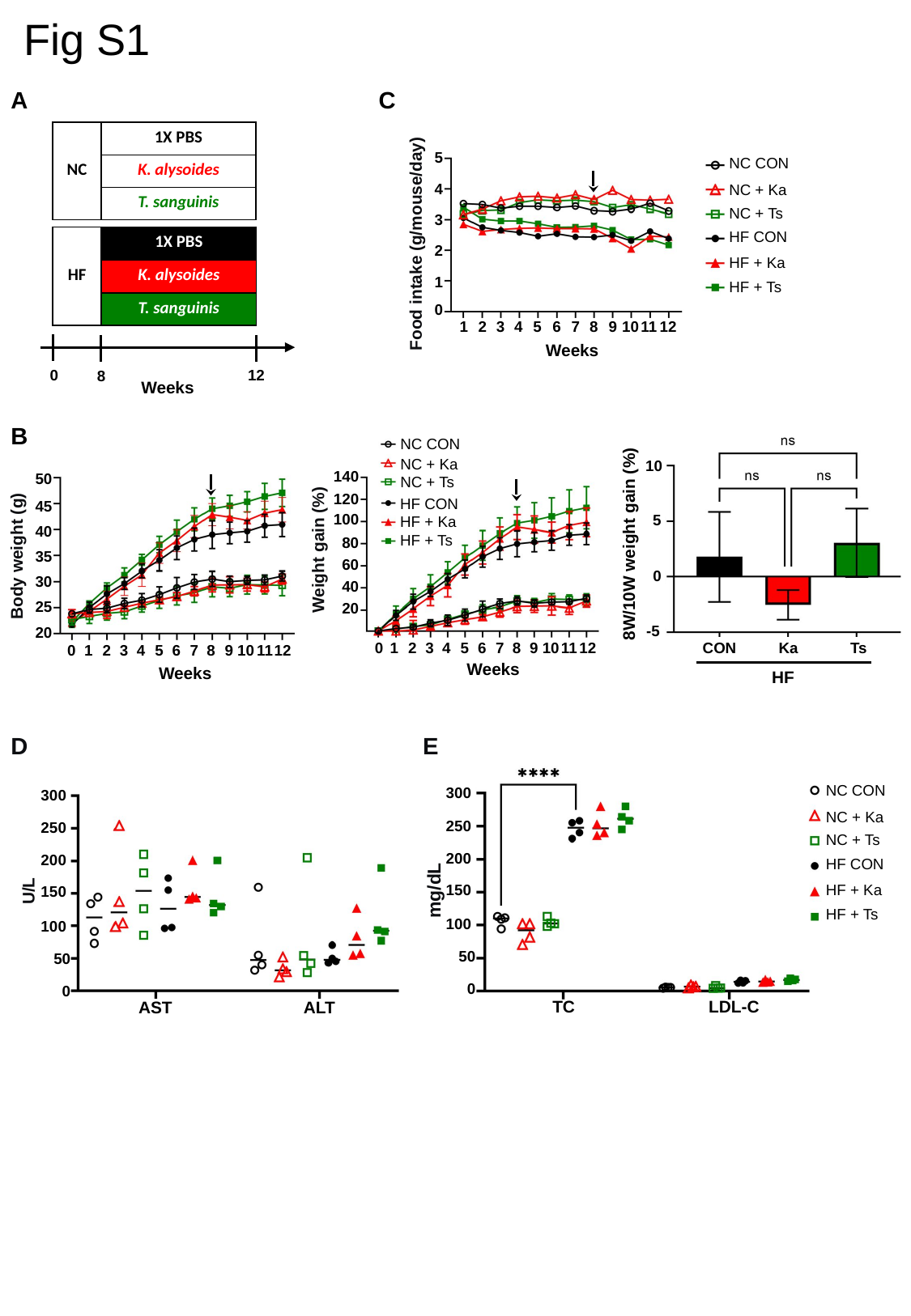

Fig S1
A
C
5
4
3
2
1
0
1
2
3
4
5
6
7
8
9
10
11
12
Food intake (g/mouse/day)
Weeks
| NC | 1X PBS |
| --- | --- |
| | K. alysoides |
| | T. sanguinis |
NC CON
NC + Ka
NC + Ts
HF CON
HF + Ka
HF + Ts
| HF | 1X PBS |
| --- | --- |
| | K. alysoides |
| | T. sanguinis |
0
12
8
Weeks
B
NC CON
NC + Ka
NC + Ts
10
5
0
-5
140
120
100
80
60
40
20
0
1
2
3
4
5
6
7
8
9
10
11
12
Weeks
Weight gain (%)
50
45
40
35
30
25
20
0
1
2
3
4
5
6
7
8
9
10
11
12
Weeks
Body weight (g)
HF CON
HF + Ka
HF + Ts
8W/10W weight gain (%)
CON
Ka
Ts
HF
D
E
300
250
200
150
100
50
0
mg/dL
TC
LDL-C
NC CON
NC + Ka
NC + Ts
HF CON
HF + Ka
HF + Ts
300
250
200
150
100
50
0
U/L
AST
ALT

## Slide 2
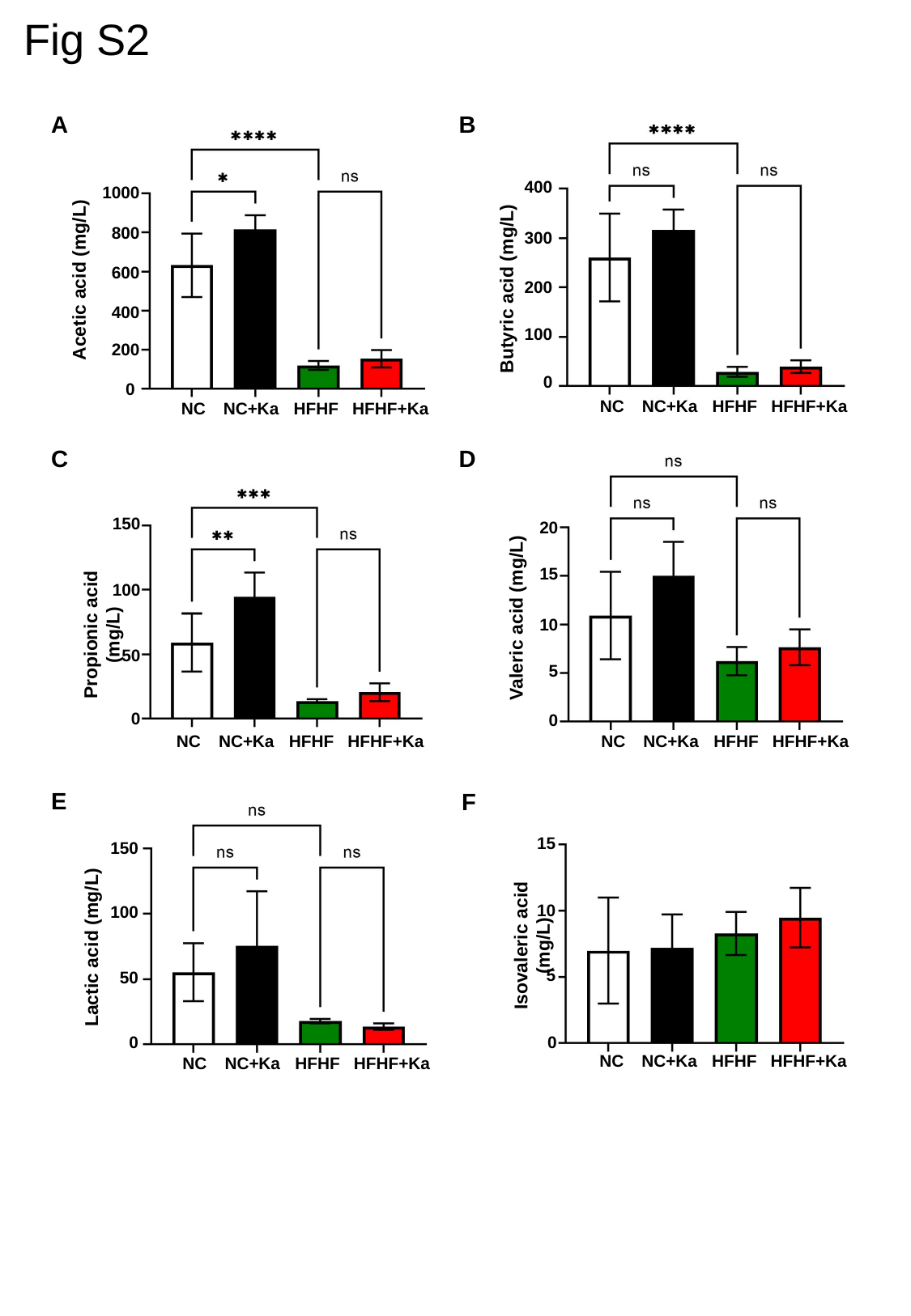

Fig S2
B
A
400
1000
800
300
600
Acetic acid (mg/L)
200
Butyric acid (mg/L)
400
100
200
0
0
NC
NC+Ka
HFHF
HFHF+Ka
NC
NC+Ka
HFHF
HFHF+Ka
D
C
150
20
15
100
Valeric acid (mg/L)
10
Propionic acid (mg/L)
50
5
0
0
NC
NC+Ka
HFHF
HFHF+Ka
NC
NC+Ka
HFHF
HFHF+Ka
E
F
15
150
10
100
Isovaleric acid (mg/L)
Lactic acid (mg/L)
5
50
0
0
NC
NC+Ka
HFHF
HFHF+Ka
NC
NC+Ka
HFHF
HFHF+Ka
