# Supplementary material for: Gut commensal Kineothrix alysoides mitigates liver dysfunction by restoring lipid metabolism and gut microbial balance
Source: Sci Rep. 2023 Sep 6;13:14668. doi: 10.1038/s41598-023-41160-y (PMC10482948; doi:10.1038/s41598-023-41160-y)
Supplement: Supplementary file 2 — Supplementary Information 2. [file 41598_2023_41160_MOESM2_ESM.docx]

**Supplementary information**

**Gut commensal *Kineothrix alysoides* mitigates liver dysfunction by restoring lipid metabolism and gut microbial balance**

Kyoung Jin Choi ^a,b^, Mi Young Yoon ^a,c^, Ji-Eun Kim ^a,d*^, and Sang Sun Yoon ^a,b,c,d,e*^

^a^ Department of Microbiology and Immunology, ^b^ Brain Korea 21 Project for Medical Sciences, ^c^ Severance Biomedical Science Institute, ^d^ Institute of Immunology and Immunological Diseases, Yonsei University College of Medicine, Seoul, Korea, ^e^ BioMe Inc. Seoul, Korea

**Supplementary information for Methods**

**Animal experiments**

As shown in Fig. S1A, the mice were fed either normal chow (NC) or high-fat (HF) diets for 8 weeks. The mice were randomly divided into six groups: NC control mice (n = 4), NC + K. a Mice fed NC and *K. alysoides* (n = 4), NC + T. s mice fed NC and *Turicibacter sanguinis* (n = 4), HF control mice (n = 4), and HF + K. a Mice fed HF and *K. alysoides* (n = 4), or HF + T. s mice fed HF and *T. sanguinis* (n = 4). PBS (control), *K. alysoides*, or *T. sanguinis* was administered to mice for 4 weeks.

**Bacterial cultivation, preparation, and treatment**

*Turicibacter sanguinis* KGMB 02607 was purchased from the Korean Gut Microbiome Bank (KGMB, Korea). Bacteria were grown at 37 °C in an anerobic chamber with mixed anaerobic gas (5% carbon dioxide, 5% hydrogen, 90% nitrogen). *T. sanguinis* was cultivated anaerobically in GAM. The GAM was deoxygenated for 24 h before use. *T. sanguinis* was then centrifuged at 8,000 rpm for 20 min and suspended in sterile 1X PBS. Mice were treated daily with 10^10^ CFU of *T. sanguinis* by oral gavage.

**Short chain fatty acids (SCFAs) quantification**

Cecal samples were collected for analyzing short-chain fatty acids (SCFAs). SCFAs, including acetic acid, butyric acid, propionic acid, lactic acid, valeric acid, and isovaleric acid, were measured using high-performance liquid chromatography on the Ultimate3000 (Thermo Dionex, USA). The cecal samples were homogenized in 5 mL of 1X PBS and filtered using a 0.22 um syringe filter (Millipore, USA). HPLC was performed using an Aminex 87H column (300 × 10 mm, Bio-Rad, Hercules, CA, USA) and RI (ERC, RefractoMAX520, Japan). SCFAs were quantified by comparison with a standard mixture (AccuStandard FAMQ-004 10 mM). The mobile phase was composed of 0.01N H_2_SO_4_ at a flow rate of 0.5 mL/min, and a measurement wavelength of 210 nm. The analysis was performed using NICEM (Seoul National University, Korea).

**Figure legends of supplementary figures**

**Figure S1. Effects of *Kineothrix alysoides* and *T. sanguinis* treatment on the NAFLD models.**

(A) Normal chow (NC)-fed mice and high-fat (HF)-fed mice were treated daily with 1X phosphate buffered saline (PBS), *K. alysoides*, or *T. sanguinis* by oral gavage for 4 weeks. (B) Body weight, weight gain percentage, and (C) food intake were measured throughout the 12-week period. (D-E) Biochemical parameters, including aspartate transaminase (AST), alanine transaminase (ALT), serum total cholesterol (TC), and serum low-density lipoprotein cholesterol (LDL-C) were measured. Statistical analysis was performed using one-way analysis of variance. **p* < 0.05; ***p* < 0.01; ****p* < 0.001; *****p* < 0.0001.

**Figure S2. Cecal short chain fatty acid (SCFA) concentrations in *Kineothrix alysoides* treated- and non-treated groups.**

SCFA concentrations in the cecal samples from each group were measured using high-performance liquid chromatography (HPLC). (A) Acetic acid (B) Butyric acid (C) Propionic acid (D) Valeric acid (E) Lactic acid (F) Isovaleric acid. Statistical analyses were performed using one-way analysis of variance. **p* < 0.05; ***p* < 0.01; ****p* < 0.001; *****p* < 0.0001.

**Table S1.** Primer sequences used for real-time PCR

| Mouse | Primer sequences |
| --- | --- |

| *SREBP1c* FWD | GGAGCCATGGATTGCACATT |
| --- | --- |
| *SREBP1c* REV | GCTTCCAGAGAGGAGGCCAG |
| *PPAR-a* FWD | CCCAAGGGAGGAATAGCTTCT |
| *PPAR-a* REV | CTCTGCGATGCGGTTCCAA |
| *PPAR-r* FWD | CGCTGATGCACTGCCTATGA |
| *PPAR-r* REV | AGAGGTCCACAGAGCTGATTCC |
| *CPT1a* FWD | TCTTCACTGAGTTCCGATGGG |
| *CPT1a* REV | ACGCCAGAGATGCCTTTTCC |
| *FABP1* FWD | TTTCAAAGGCATAAAGTCCGTG |
| *FABP1* REV | CTTGCTGACTCTCTTGTAGACA |
| *CD36* FWD | TTGTACCTATACTGTGGCTAAATGAGA |
| *CD36* REV | CTTGTGTTTGAACATTTCTGCTT |
| *ZO-1* FWD | AAAAGTGAACCACGAGATGCT |
| *ZO-1* REV | AAAGGTGGAGGACTGGAGATGA |
| *Occludin* FWD | ACCCGAAGAAAGATGGATCG |
| *Occludin* REV | CATAGTCAGATGGGGGTGGA |
| *Claudin-3* FWD | CACCACTACCAGCAGTCGATGAAC |
| *Claudin-3* REV | AGACTGTGTGTCGTCTGTCACCATC |
| *TNF-a* FWD | TAGCCAGGAGGGAGAACAGA |
| *TNF-a* REV | TTTTCTGGAGGGAGATGTGG |
| *IL-1b* FWD | TTGAAGAAGAGCCCATCCTC |
| *IL-1b* REV | CAGCTCATATGGGTCCGAC |
| *GAPDH* FWD | GGCATTGCTCTCAATGACAA |
| *GAPDH* REV | ATGTAGGCCATGAGGTCCAC |
